# The philosophy of psychiatry and biologism

**DOI:** 10.3389/fpsyg.2014.01032

**Published:** 2014-09-18

**Authors:** Marco Stier, Bettina Schoene-Seifert, Markus Rüther, Sebastian Muders

**Affiliations:** ^1^Institute for Ethics, History and Theory of Medicine, University of MuensterMuenster, Germany; ^2^Ethics in the Neurosciences, Institute of Neuroscience and Medicine, Forschungszentrum JülichJülich, Germany; ^3^Department of Philosophy, Center for Ethics, University of ZurichZurich, Switzerland

**Keywords:** ethics, medical, biologism in psychiatry, reductionism, psychiatry, philosophy of neuroscience

In the philosophy of psychiatry, there has been an ongoing dispute about the capabilities and limits of the bio-natural sciences as a source of methods and knowledge for quite some time now. Still, many problems remain unsolved. This is at least in part due to the regrettable fact that the opposing parties are far too rarely prepared to swap ideas and to try to increase their mutual understanding. On the one hand there are those—psychiatrists as well as philosophers—who maintain a more mentalistic and/or phenomenalistic view of the psyche and its disturbances. On the other hand there are researchers who follow biologically inspired strategies: Since the human mind is something through and through biological, mental diseases, too, can and should be explained and treated biologically. Even though there are examples of fruitful collaboration, in general the split prevails. One often gets the impression that both sides remain in their “trenches”, busy with confirming each other's opinions and developing their positions in isolation. Even though there are also examples of fruitful collaboration, the split leads to several shortcomings:

1. Good arguments and insights from both sides of the debate get less attention than they deserve.
2. The further improvement of each position becomes harder without criticism, genuinely motivated by the opposing standpoint.
3. The debate is not going to stop, at least not in the way it would finish after a suggested solution finds broad support.
4. Related to this, insisting on the ultimate aptness of one side is just plainly wrong in almost every case, since undeniably, most philosophical positions usually have a grain of truth hidden in them.

In sum, many controversies persist with regard to the appropriate methodological, epistemological, and even ontological level for psychiatric explanation and therapies. In a conference which took place in December 2011 in Muenster, Germany, we tried to contribute to a better understanding about what really is at issue in the philosophy of psychiatry. We asked for a possible common basis for several positions, for points of divergence, and for the practical impact of different solutions on everyday work in psychiatry.

The present Frontiers research topic is a fruit of that conference. Since psychiatry is a subject too wide to be covered *in toto*, this research topic collects six target articles, each focusing a particular aspect. They are accompanied by a number of commentaries providing both critical and supportive arguments.

First, Henrik Walter sets the stage by presenting what he calls “the third wave of biological psychiatry” (Walter, 2013). The first two waves were primarily driven by the ambition to uncover the relation between mind and brain and by the integration of genetic insights as well as the upcoming of psychopharmacology. While these where—in a sense—one-sided, the third wave, starting only in the last two decades of the previous century, conceptualizes mental disorders “as brain disorders of a special kind.” As Walter explains, they require “a multilevel approach ranging from genes to psychosocial mechanisms.” This broader account might be an indication that the alleged reductionism today's biological psychiatry is often accused of is unjustified. Markus Pawelzik doubts in his commentary that the “third wave,” as conceived by Walter, has in fact the potential to overcome psychiatry's biologistic thought (Pawelzik, 2013). Michael Noll-Hussong picks up on Walters idea of “waves,” arguing that the “sinks” between them have to be taken into account, too, for an adequate understanding of psychiatry's momentum (Noll-Hussong, 2014). He predicts the upcoming of a fourth wave that will arise from the background of information integration theory, using computer-simulations of the mind to increase our understanding of the psyche. In a third commentary Gerhard C. Bukow cautions against using externalist approaches of mental disorders too uncritically (Bukow, 2013). What externalist accounts need, but hardly can provide, are criteria where to stop adding further and further external constituents to the notion of psychiatric disorder.

In the second target article Marco Stier argues against the reducibility of the concept of mental disorder (Stier, 2013). Even if the mental may in principal be reducible to brain functions, mental disorders are not, Stier holds. The reason is that we can only call behavior disordered by comparing it to non-disordered behavior, i.e., by using norms which, in turn, are not reducible to anything physical. Stier's claim has provoked a number of critical replies. Markus Rüther finds several argumentative shortcomings in Stier's account (Rüther, 2014). According to Rüther, Stier's constructivist thesis and the associated anti-reductionism suffer from a lack of argumentative force. Similarly, Sebastian Muders in his comment examines the relation between normativity on the one hand, and non-naturalness, non-objectivity, and relativity, on the other (Muders, 2014). He argues that the normative character of mental disorders does not mean that they are non-natural, non-objective, or relative. Anneli Jefferson principally agrees with Stier, but adds two aspects regarding normativity and non-reducibility: she holds firstly that the importance of the mental level can be explained independently of value judgments, secondly she points out the need to investigate normativity in any kind of disease or disorder ascriptions, not just in the mental area (Jefferson, 2014). Bettina Schoene-Seifert stresses—in accordance with Jefferson—that values don't come into play only in regard of mental disorder (Schoene-Seifert, 2014). Above that, she warns against mistaking Stier's argument as being one in favor of methodological antireductionism in psychiatry. Peter Hucklenbroich's commentary is on both Stier and Walter, bringing central and well established principles of modern medical pathology to mind (Hucklenbroich, 2014). In particular, he opposes the normativity claim, arguing that the criteria of pathologicity are rooted in nature and not relative to social norms and values.

The third target article by Thomas Schramme is concerned with the autonomy of the concept of disease in psychiatry (Schramme, 2013). On the background of classical ideas from the philosophical debate on the mind-body problem, he argues that denying substance dualism does not force us to adopt a purely materialistic account of the mental. Especially, some psychiatrists' belief that this denial necessarily leads to a neurophysiological account of mental disorder is wrong in his eyes. Even in the absence of any form of substance dualism we still need an irreducible psychological level of explanation. Marcella Rietschel argues in her commentary, contra Stier and Schramme, that mental disorders in actual fact are somatic disorders, as actual scientific insights show (Rietschel, 2014). According to Jan-Hendrik Heinrichs the terms “psychiatric disorder” and “neural defect” belong to different types of analysis and cannot be identified with or reduced to each other (Heinrichs, 2014). While Schramme argues on a more general level, Michael Jungert takes up his view and exemplifies the irreducibility of the mental on the basis of posttraumatic stress disorder (Jungert, 2013). Since an analysis of the internal perspective of a patient is indispensable, neither neuroscience nor any biological psychiatry is able to approach mental disorders appropriately. The final comment in this section is again on Stier and Schramme together. Gerald Ulrich stresses that the currently prevailing “either-or interrogations” are utterly ill-posed (Ulrich, 2014). In the context of aspect dualism regarding mind and body Ulrich recommends to realize that what is at stake is an “as-well-as” issue.

One of the central criticisms against biological accounts in psychiatry is that they disregard the phenomenal perspective of the suffering person. The target article by Kerrin A. Jacobs focuses on this aspect by analyzing “the depressive situation” (Jacobs, 2013). Her approach is a phenomenological one, but one that is informed by empirical research. In depression, she explains, the pre-reflective self-evaluative dynamics of the depressed is significantly altered, leading to impairments of agency. While Jacobs stresses the *pre*-reflective dimension of depression Lara Rzesnitzek in her commentary reminds of the importance the notion of a “self-feeling” had in early theories of depression (Rzesnitzek, 2014). Although her commentary is a “historical note” it nevertheless points to one of the most paramount problems in debates on current biological psychiatry.

The article of Hanfried Helmchen, an experienced practicing psychiatrist, cautions against any dogmatism in psychiatry (Helmchen, 2013b). As history shows, an exclusively biological account of mental disorder is as disadvantageous for the patient as an exclusively social or psychological one. What is needed instead is a biopsychosocial model of mental disorder. In his commentary Marco Stier admits that the integrative account favored by Helmchen is indeed important as a warning sign against misapprehensions of the mental (Stier, 2014). But he assumes nevertheless that it will in effect either lead to explanatory arbitrariness, or end up as a theory that is ultimately biological.

Last not least, the target article by Lara Rzesnitzek shows that some issues debated in today's psychiatry have already an astonishing long history (Rzesnitzek, 2013). An example of this is the “psychosis risk syndrome”—one of the contentious points in the preparation of the fifth revision of the Diagnostic and Statistical Manual of Mental Disorders (DSM-5). Even though attempts of identifying symptoms of a looming schizophrenia are much older, in 1938 “early psychosis” entered the stage as a possible independent diagnosis. Hanfried Helmchen comments on ethical implications of “early psychosis,” and adds some remarks to Rzesnitzek's historical description (Helmchen, 2013a). Finally, Nicolas Henckes suggests complementing this historical picture with a sociological view (Henckes, 2014). As he points out, psychiatric diagnoses always have a life of their own outside medicine. This is especially true for politics and the people affected.

Our approach to the research topic “philosophy of psychiatry and biologism” is not intended to be exhaustive. Rather, our aim was to bring together experts of different fields in order to work with—and not against—each other. In this sense, the articles and commentaries of the present volume may serve as a stepping-stone for future cooperation.

Our work on biologism in psychiatry was part of a research fellowship of Bettina Schoene-Seifert at the Max-Planck-Society. The respective project was entitled “Do the life sciences threaten human self-understanding? Analyzing current debates between sciences and humanties.” We hereby want to express our gratitude to the Max-Planck-Society for having made possible the whole project.

## Conflict of interest statement

The authors declare that the research was conducted in the absence of any commercial or financial relationships that could be construed as a potential conflict of interest.
